# Altered Tuber Yield in Genetically Modified High-Amylose and Oil Potato Lines Is Associated With Changed Whole-Plant Nitrogen Economy

**DOI:** 10.3389/fpls.2018.00342

**Published:** 2018-03-15

**Authors:** Fereshteh Pourazari, Mariette Andersson, Martin Weih

**Affiliations:** ^1^Department of Crop Production Ecology, Swedish University of Agricultural Sciences, Uppsala, Sweden; ^2^Department of Plant Breeding, Swedish University of Agricultural Sciences, Alnarp, Sweden

**Keywords:** *Solanum tuberosum*, amylose potato, oil potato, genetically modified plants, nitrogen use efficiency, yield, field experiment, greenhouse experiment

## Abstract

Breeding for improved crop quality traits can affect non-target traits related to growth and resource use, and these effects may vary in different cultivation conditions (e. g., greenhouse vs. field). The objectives of this study are to investigate the growth and whole-plant nitrogen (N) economy of two genetically modified (GM) potato lines compared to their non-GM parental varieties and when grown in different cultivation conditions. A high-amylose GM potato line and its parent were grown under field and greenhouse conditions for one growing season in Sweden; and a GM oil potato line and its parent were grown in greenhouse conditions only. Tuber yield, above ground biomass, N uptake efficiency and other plant N economy traits were assessed. In both cultivation conditions, the GM lines produced between 1.5 and two times more tubers as compared with their parents. In the greenhouse, fresh tuber yield and N uptake efficiency were unaffected by the genetic modifications, but the GM-lines produced less tuber biomass per plant-internal N compared to their parents. In the field, the fresh tuber yield was 40% greater in the high-amylose line as compared with its parent; the greater fresh tuber yield in the high-amylose GM line was accomplished by higher water allocation to the harvested tubers, and associated with increased N recovery from soil (+20%), N uptake efficiency (+53%), tuber N content (+20%), and N accumulation (+120%) compared with the non-GM parent. The cultivation conditions influenced the yield and N economy. For example, the final fresh above-ground plant biomass and N pool were considerably higher in the greenhouse conditions, whilst the tuber yield was higher in the field conditions. In conclusion, the genetic modification inducing high accumulation of amylose in potato tubers affected several non-target traits related to plant N economy, and increased the plant N uptake and accumulation efficiency of the field-grown plants. Due to strongly increased plant N accumulation compared to the parental variety, the cultivation of the high-amylose line is expected to require higher N fertilization rates. However, starch productivity per unit land area or soil N still is expected to be higher in the high-amylose line.

## Introduction

Biotechnology can be used to modify specific traits, for example, to improve yield and quality in crops. However, due to physiological and genetic links between these traits (Stearns, 1989; Weih, 2003), modification of the crop's specific traits with conventional or biotechnological methods may also be associated with effects on non-target physiological traits and processes. The modification of a crop's functional traits can influence crop characteristics that in turn affect the crop production system, e.g., in terms of resource use. That is to say that the individual traits of plants can greatly impact ecosystem processes (de Bello et al., 2010; Kolseth et al., 2015). Therefore, in order to evaluate the potential agro-ecological consequences of trait modifications in newly developed crops (Lau et al., 2014), a quantitative assessment of resource use and growth pattern in for example genetically modified (GM) crops in comparison to the related non-GM parental varieties is crucial. Furthermore, when assessing the safety of GM crops, substantial equivalence studies, comparing GM lines and their parents, are frequently applied. It should be kept in mind though, that external influences, like the environmental conditions during growth, can affect plant metabolism, as was shown in a metabolite study of field-grown wheat (Baker et al., 2006).

Starch is an important agricultural product and is widely used for food as well as for industrial raw material (Birch et al., 2012). In this context, high-amylose starch has received great attention in the last decades for, among other end uses, yielding a low glycemic index when consumed and for delivering a preferable quality as feedstock for packaging material (Richardson et al., 2000; Tuck et al., 2006). Potato (*Solanum tuberosum* L.) is a high yielding crop which is widely used for starch production; particularly in northern Europe. Starch from high-amylose potato lines, which can be developed by inhibiting two starch branching enzymes, is favorable for film-forming and bioplastics (Menzel et al., 2015). Induction of oil accumulation in potato tubers enhances the nutritional content of a starchy staple crop; and can be achieved by introducing a transcription factor that triggers lipid biosynthesis. The oil accumulation in tubers can also give an added value to potatoes grown for the starch industries, yielding an additional commercial product to be recovered during starch production (Hofvander et al., 2016). Starch and oil potatoes are highly relevant alternatives in the context of bio-economy, which is of outmost importance to solve some of the greatest global challenges we are facing today. In this context, the future bio-economy will depend on advanced technology, cost effectiveness and sustainable biomass availability (Scarlat et al., 2015). Oil- and starch crops are key sources for a bio-economy relying on the sustainable production of biomass, and plant pre-breeding research to enhance crop yield (e.g., starch content) output in relation to the resource (e.g., N fertilizer) input is under constant focus in these crops (Zeeman et al., 2010; Yadava et al., 2012). To evaluate and compare different crops generating the same type of biomass, the methods used for the assessment of biomass yield in relation to a limiting resource (e.g., N fertilization) need to be applicable across a wide range of different crops.

A changed carbon metabolism due to induced accumulation of amylose and oil may involve re-direction of plant internal carbon flows and allocation, due to physiological links between the traits. For example, in the studies by Hofvander et al. (2004) and Menzel et al. (2015), higher amylose concentration in potatoes was associated with a lower starch content and higher fresh tuber yield. Similar results, with low starch content and increased tuber yield, have been documented in other potato studies considering various traits (Müller Röber et al., 1992; Tjaden et al., 1998; Riewe et al., 2008).

Thus, altered carbon allocation in GM potato lines may also influence the economy of growth-limiting resources such as nitrogen (N). As for most agricultural crops, the availability of N is one of the most important growth-limiting factors in potato production (Zotarelli et al., 2015). However, high N fertilizer demand, along with a generally poor exploitation capacity in potato plants due to their shallow root system (Iwama, 2008), is often associated with negative environmental impacts such as N leaching from the soil (Sharifi and Zebarth, 2006). Due to the environmental risks and high economic costs associated with N fertilization in agriculture, the identification of any crop characteristic that can potentially influence the crop N utilization and N cycling at the ecosystem scale is important. Since crop functional traits (e.g., traits affecting crop N utilization) may greatly influence the production system, a quantitative comparison of potato growth and N economy between GM lines and their parents is of great importance.

In general, plant N economy addresses the properties of a plant in terms of the production of biomass or yield in relation to the accumulation and use of N. Thus, N economy focuses on the plant characteristics improving its efficiency with respect to the use of N. The frequently used term N use efficiency (*NUE*) may refer to the properties of either a plant or a production system with respect to the use of N, and can be assessed with different methodologies (Fageria and Baligar, 2005; Reich et al., 2014). In this study, the potato N economy was evaluated using two different approaches: (a) the plant- and soil-based *NUE* concept by Moll et al. (1982), separating N uptake or recovery efficiency from the soil (*NUpE*) and N utilization efficiency (*NUtE*) as the components of *NUE*; and (b) the plant-based approach by Weih et al. (2011), quantifying the plant-internal nutrient accumulation efficiency (*NAE*, called *NUE* in the original publication) and its components. The *NAE* by Weih et al. (2011) is further broken down into three components: (1) N uptake efficiency (*U*_*N*_), which is the ratio between mean plant N content during the growth period and N in the initial biomass (here, planted potato cuttings or seed tubers); (2) yield-specific N efficiency (*E*_*N,yield*_), which is the ratio between desired yield (here, tubers) and the mean plant N content during the growth period; representing the efficiency of converting accumulated N into tuber biomass; and (3) yield N concentration (*C*_*N,yield*_) or tuber N content, which represents the efficiency of N re-translocation into the harvested tubers. The *U*_*N*_ is similar to the *NUpE* (Moll et al., 1982), but does not use the soil N content per unit area as the basis for the quantification of N uptake efficiency. Any estimate of the plant available soil N per unit area is heavily depending on the methodology of soil N analysis and also an identity with poorly defined system boundaries, whereas the initial plant N pool used for the calculation of *U*_*N*_ is less problematic in terms of the N analysis methodology and has well defined system boundaries (Weih et al., 2017). In contrast to *NUpE*, the *U*_*N*_ integrates plant N pools during the entire growing season and therefore needs to consider also the initial N pool, e.g., in the seed, which is essential for early plant growth and establishment (Liptay and Arevalo, 2000) despite of its often small quantity. Also, the *E*_*N,yield*_ is similar to the *NUtE* (Moll et al., 1982), but does not rely on the plant N pool at only one single developmental stage as the basis for its calculation. Instead, the calculation of *E*_*N,yield*_ is based on the mean plant N pool during the entire period of yield generation, which appears a better estimate than the N pool of a single growth stage especially for potato, producing tubers during nearly the entire growing season. In summary, both the *NUE* and its components (Moll et al., 1982) and the *NAE* and its components (Weih et al., 2011) are here used to inform about genotypic variation in various aspects of crop N accumulation (affecting N fertilization requirements) and N economy aspects in relation to the tuber productivity.

The overall aim of this study was to explore the influence of genetic modification for development of high-amylose starch and induced oil synthesis in potato tubers on plant growth and N economy, and to document the effects when grown under different cultivation conditions. The objectives were (i) to evaluate the yield and whole-plant N economy (*sensu NUE, NAE* and their components) of two GM potato lines compared with their non-GM parents; and (ii) to explore the influence of two different cultivation environments (greenhouse and field). Accordingly, we explored the hypotheses that (i) the high-amylose and oil GM potato lines have greater tuber yield compared with their non-GM parental varieties; (ii) the higher tuber yield in the GM potato lines is associated with a higher N uptake efficiency, N utilization efficiency and/or yield-specific N efficiency; and (iii) the effect of modified quality traits on the non-targeted traits is similar in different growth conditions (field and greenhouse conditions).

## Materials and methods

### Plant material

Two potato (*Solanum tuberosum* L.) lines, genetically engineered for high-amylose starch content and for oil accumulation in the tubers were compared to their non-GM parental varieties. “Dinamo” is the parental variety to “T-2012,” a GM line for high-amylose starch; and “Kuras” is the parental variety to “T-8003,”a GM line for oil accumulation in tubers. Both the high-amylose line and oil potato line were developed in studies described elsewhere (Menzel et al., 2015; Hofvander et al., 2016). The oil (here, triacylglycerol; TAG) content was 0.5 and 7.0 nmol TAG per mg tuber dry matter in “Kuras” and “T-8003,” respectively (Hofvander et al., 2016). The amylose content was 23 and 49% of total starch for “Dinamo” and “T-2012,” respectively (Menzel et al., 2015).

### Field experiment

The field experiment was conducted in Borgeby, southern Sweden, (55° 59′ N, 13° 35′E) during the period May to October 2015. The mean air temperatures and accumulated precipitation for the experimental period and for a long-term period (2007-2014) are presented in Table 1. Seed tubers of the potato line “T-2012” and its parental variety “Dinamo” were planted on 7th May in four rows; with 0.75 m spacing and 0.32 m distance between the plants on the rows. To avoid edge effects, the rows were placed in the middle of a larger experiment with a similar potato plant density. In total, 15 potato seed tubers from “T-2012” and “Dinamo” were planted in each row, consisting of five randomly placed groups of three plants from each line. The field trial was conducted with permission from the Swedish Board of Agriculture, Jönköping, Sweden. The soil was loamy sand (FAO, 2015) at pH 6.4. The mean total P and K in the soil were 0.16 and 0.08 g kg^−1^, respectively (ammonium lactate method, air-dried soil). The plants were irrigated with about 30 mm of water approximately every 12 days starting from July 2nd. Before planting, fertilizer solution was applied corresponding to 1.8, 0.8, 3.0 g plant^−1^ of N, P, and K, respectively (NS 27-4 Axan., Yara AB, Malmö, Sweden) On 2nd July, 2.5 g N together with 0.4 g S plant^−1^ were applied to the plots. Given that approximately four plants were grown per m^2^, a total amount of 17.7 g N m^−2^ (corresponding to 177 kg N ha^−1^) was applied to the plants, while the total plant N content at the final harvest varied between 13.7 and 16.7 g N m^−2^. The plants were treated with pesticides in accordance with the local recommendation for starch potato production.

**Table 1:** Mean monthly temperature and total precipitation in the period May to October during the field experiment in 2015, compared to long term means (2007–2014) for the experimental site, Borgeby, Sweden (data source: http://www.slu.se/faltforsk).

	**Temperature (°C)**		**Precipitation (mm)**	
	**2007–2014**	**2015**	**2007–2014**	**2015**
May	12.1	10.4	156	163
June	15.3	13.8	200	198
July	18.1	17.6	285	251
August	17.4	18.0	398	301
September	13.9	13.7	438	355
October	9.4	9.2	501	369

### Greenhouse experiment

The greenhouse experiment was conducted during September–December, 2014 in Alnarp, southern Sweden. In the greenhouse, the day light was set to 16 h day^−1^ with supplementary lighting (photon flux ~200 μmol s^−1^ m^−2^); the relative humidity was 60% and the temperature was set to 18/15°C for day/night (temperatures above 18°C might have occasionally occurred). The experiment was a completely randomized block design with five replicates. The experimental units were 7.5 L plastic pots and a single potato plant was grown in each pot. Four potato lines including two parental varieties (“Kuras” and “Dinamo”) and two GM lines (“T-8003” and “T-2012”) were grown in the pots. From each potato line, 20 *in vitro* propagated clones were planted as cuttings on 17th September in the pots (80 pots in total). Soil consisted of a mixture of light and black peat and sand at pH 6 (yrkesplantjord, SW Horto, Hammenhög, Sweden). The mean total nitrogen (N), phosphorous (P), and potassium (K) in the soil were 1.8, 0.9, and 1.9 g kg^−1^, respectively. Fertilizer, SW Bouyant RikaS 7-1-5+mikro (SW Horto, Hammenhög, Sweden) was applied once a week, starting from the second week after planting. A standard nutrient mix (N 10.2, P 2.0, K 8.6, Mg 0.8, Ca 0.6, Mn 0.04, Fe 0.07, Cu 0.003, Zn 0.006, B 0.02, Mo 0.0008; concentrations in g L^−1^) was diluted in water and applied directly into the pots. The fertilizer was applied with increasing amounts as plants grew larger and with decreasing amounts toward the end of the growth period, i.e., 0, 10, 50, 50, 150, 150, 300, 300, 300, 200, 200, 100, 100, 50, 0 mg N pot^−1^, respectively, during the weeks 1–15 after planting. In total, fertilizer was applied 13 times and the N application ranged between 0.01 at the establishment phase and 0.30 g N pot^−1^ application^−1^ at flowering time (technically corresponding to ~2–61 kg ha^−1^). Therefore, the total N fertilizer applied to each potato plant was 1.96 g N pot^−1^ technically corresponding to ~400 kg N ha^−1^. In order to reduce the growth-limiting effect of nutrients other than N, the other nutrients were applied in their corresponding proportions. The pots were placed a few centimeters from each other on a table. The small plantlets were gently irrigated from above, while the established plants were watered from below by shallowly flooding the table for 1.5–2 h 2 or 3 times a week. Potato plants were treated against thrips by application of a biological pest control measure; i.e., *Amblyseius swirskii* predatory mites (Koppert B.V, Berkel en Rodenrijs, the Netherlands) were introduced using slow-releasing bags. Tubers from 5 pots of “T-2012” and “Dinamo” harvested in December were stored at 8°C for 4 months and used as seed potatoes for the field experiment.

### Measurements and sampling

The sampling in both experiments took place at the following developmental stages based on the BBCH-scale (Biologische Bundesanstalt und CHemische Industrie) of potato plants, given by Hack et al. (1993): At tuber initiation (H1, BBCH 29; 2nd October in greenhouse, 1st July in field), at flowering (H2, BBCH 65-69; 12th−14th November in greenhouse, 20th August in field) and at maturity (H3, BBCH 95; 16th−19th December 2014 in greenhouse, 19th October in field). The sampling of biomass and N contents at these stages enabled us to later calculate estimates of the mean plant N pool during the growing season, which is required in the *NAE* concept (Weih et al., 2011). The final harvest at tuber maturity stage was performed according to the local harvest time for potato, i.e., when about 50% of the above-ground plant parts have reached senescence (Hack et al., 1993). In the greenhouse at each harvest, the above-ground biomass and the root and tubers biomass of five potato plants were harvested from each line. The above-ground plant parts, potato tubers and the underground stems and roots were separated for each individual plant. The roots and below-ground stems were carefully separated from the soil and rinsed from dirt with tap water for below-ground biomass assessments. In the field at each harvest occasion, the tuber and above-ground biomass of 10 potato plants from each line were harvested. The above-ground biomass was sampled from surface level; and the underground stems that reached the light and had active leaf area were also harvested as above-ground parts. At each harvest, the fresh and dry weight for tuber and above-ground biomass, and the number of tubers were assessed. In both experiments, the greenness or relative chlorophyll concentration of leaves was measured on three leaves along the stem of 10 plants per potato line at flowering (i.e., H2), using a SPAD-502 leaf chlorophyll meter (Konika Minolta Sensing, Japan) (Uddling et al., 2007). The averages of the three SPAD values collected from each plant were used for data analysis.

Collected above-ground and tuber samples from both experiments were cut into smaller pieces and oven dried (Heratherm OGS400, Thermo Scientific, USA) at 80°C for 3–7 days depending on the amount of biomass. The dried plant materials were weighed for biomass assessments and ground using a knife mill, thereafter with a ball mill (Retsch GmbH & Co. KG, Haan, Germany). The N concentration was obtained for above-ground biomass from all the harvests, and from dry tubers at H2 and H3. The N analysis was carried out on a LECO CNS-2000 automated dry combustion analyzer (LECO, St. Joseph, MI) using a standard method (SS-ISO13878).

### Assessment of plant nitrogen economy

The *NUE* and its components, i.e., N uptake or recovery efficiency from soil (*NUpE*) and N utilization efficiency (*NUtE*), were assessed by using the approach by Moll et al. (1982) only in the field experiment. Accordingly, the *NUE* is defined as the ratio of final tuber yield (after vine desiccation) to soil N per area, which is the sum of the N in the soil before fertilization and the applied N in the fertilizer. According to the original definition by Moll et al. (1982) for cereals and also the application to potato by Zebarth et al. (2004), the *NUpE* is the plant N pool at maturity (at H3 in our study) divided by the soil N per area; and the *NUtE* is the yield (here tuber) divided by the N pool at maturity. We also calculated the amylose yield per unit N in the plant (*NUtE*_*amylose*_) and the soil (*NUE*_*amylose*_) to evaluate the N utilization and use efficiencies in terms of the targeted end product.

Both in the greenhouse and field experiment, the whole-plant N accumulation efficiency (*NAE*) and its components, i.e., N uptake efficiency (*U*_*N*_), yield specific N efficiency (*E*_*N,yield*_) and yield N concentration (*C*_*N,yield*_) (i.e., tuber N content), were assessed according to Weih et al. (2011); all calculations of *NAE* and its components are presented in Table 2. Accordingly, the overall *NAE* is the final N yield divided by the N content in the initial plant material, and thus the ability of crops to multiply the N available in the initial seed (i.e., cuttings in the greenhouse experiment and seed potato in the field experiment). The mean plant N content in the biomass during the entire growth period (*N'*, Table 2) was assessed based on the mean plant N content (including tubers and above-ground plant parts) at different developmental stages, by taking into account the different lengths of the corresponding periods of time. Thus, *N'* was calculated based on the periods between the tuber initiation and flowering stages (H1–H2), and between flowering and tuber maturity stages (H2–H3).

**Table 2:** Calculation of whole-plant N use efficiency (*NAE*) and its components based on Weih et al. (2011), and the acronyms used in this manuscript.

**Symbol (Unit)**	**Component**	**Calculation**
*N_*S*_* (g plant^−1^)	N content in the initial seed (cutting or seed potato)	
*N'* (g plant^−1^)	Mean plant N content during entire growth period	((*N_*mean*(*H*1−*H*2)_ × t_*(*H*1−*H*2)*_*) + (*N_*mean*(*H*2−*H*3)_ × t_(*H*2−*H*3)_*))/*t_(*H*1−*H*3_*_)_
*N_*mean(*H*1−*H*2)*_* (g plant^−1^)	Mean plant N content during H1 and H2	
*N_*mean(*H*2−*H*3)*_* (g plant^−1^)	Mean plant N content during H2 and H3	
*t_*(*H*1−*H*2)*_* (Day)	Period between H1 and H2	
*U_*N*_* (g g^−1^)	N uptake efficiency	*N'*/*N_*S*_*
*E_*N,yield*_* (g g^−1^)	Yield specific N efficiency	*DB_*Tuber*_*/*N'*
*C_*N,yield*_* (g g^−1^)	Yield N concentration	*N yield*/*DB_*Tuber*_*
*NAE* (g g^−1^)	N accumulation efficiency	*U_*N*_* × *E_*N,yield*_* × *C_*N,yield*_*
*DB_*Tuber*_* (g plant^−1^)	Tuber dry biomass at final harvest	
*FB_*Tuber*_* (g plant^−1^)	Tuber fresh biomass at final harvest	
*B_*Ag*_* (g plant^−1^)	Dried above-ground biomass at final harvest	

*H1, H2, and H3 represent the first, second and third harvests, respectively*.

### Statistical analysis

Analysis of variance was carried out separately for the field and greenhouse experiment, using the mixed procedure, SAS version 9.3. For the greenhouse experiment, the statistical analysis was performed using a hierarchical mixed ANOVA model by including the related GM and parental varieties nested within the potato lines. In the ANOVA tables, the “within line types” effect refers to the comparisons between the GM lines and their parental varieties, and the “between lines types” effect in the ANOVA tables shows the comparisons between the two groups of oil and amylose potato lines (an effect which was not in the focus of this paper, but still is presented in the tables). In the field experiment, the potato “line” was treated as a fixed effect and block as a random effect. The homogeneity of variance and normality were examined prior to the statistical analysis. Because the residuals were not normally distributed in tuber fresh and dry biomass, *NAE* and *C*_*N,yield*_, those values were first log transformed and the estimated mean and standard deviation values were back-transformed.

## Results

### Field experiment

**Figure 1:**
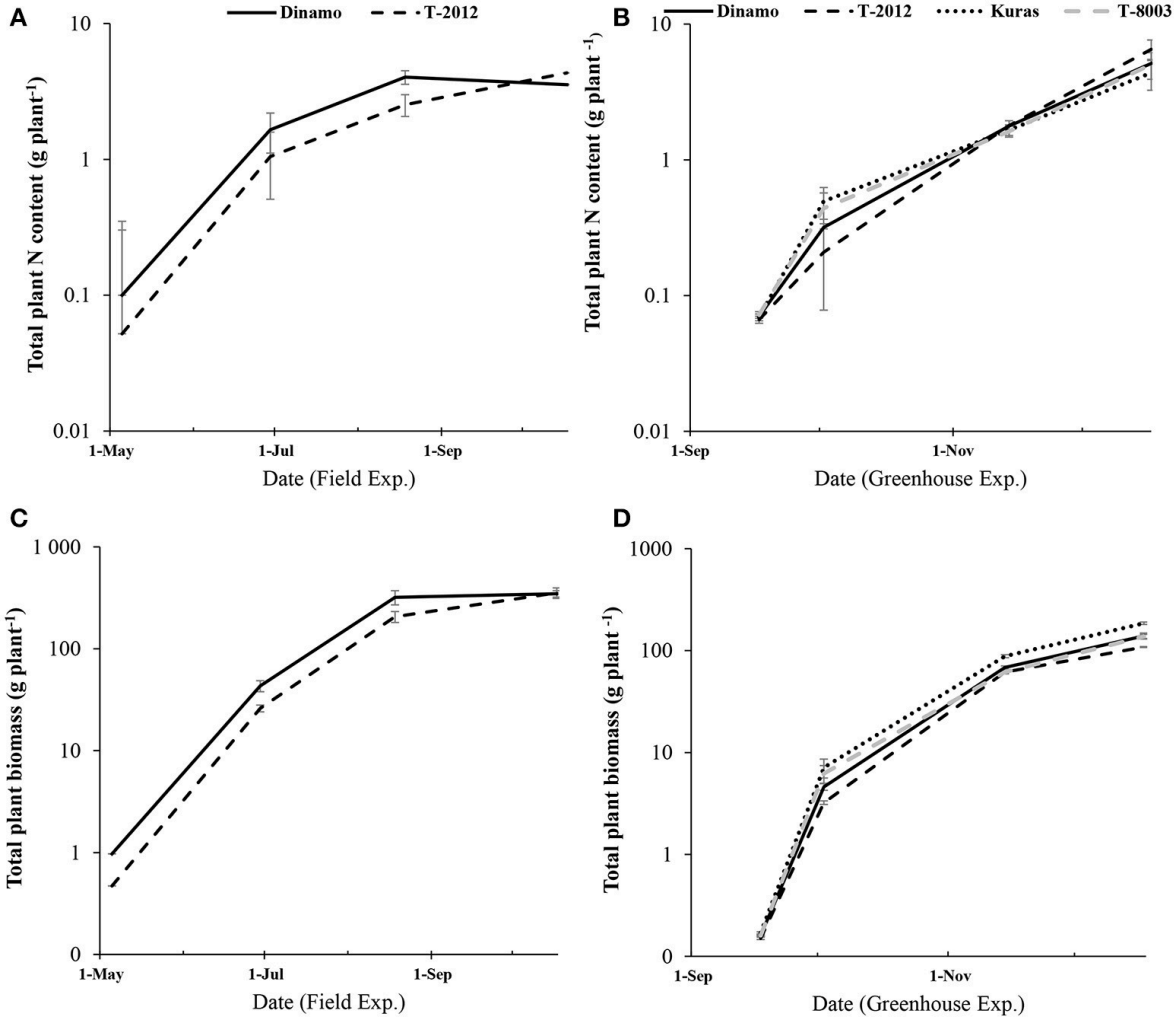
Development of total plant N content and biomass accumulation (in above-ground and tubers) during two experiments from the initial biomass to tuber initiation, flowering stage and final harvest. **(A,C)** the field study (May to October; 2015) and **(B,D)** the greenhouse experiment (September- December; 2014). Note the logarithmic scale in the y-axis. The bars are standard errors (*n* = 5 and 10 in greenhouse and field, respectively).

Only the high-amylose potato line “T-2012” and its parental variety “Dinamo” were grown in the field. The total N content in the initial seed tubers (*N*_*S*_) was twice as high in “Dinamo” than “T-2012,” due to a higher individual tuber weight in “Dinamo” (Figure 1; Table 3). The above-ground biomass of “Dinamo” was nearly 50% greater than “T-2012” when observed at tuber initiation, whereas at the flowering stage, both lines had similar above-ground biomass and fresh tuber biomass per plant (Table 4). At the flowering stage, the total tuber dry biomass per plant was 63% higher in “Dinamo” than the GM line “T-2012” (Table 4).

**Table 3:** Mean of plant N in the initial seed, total plant N at tuber initiation stage (H1), flowering stage (H2) and final harvest (H3), mean N in the above-ground biomass (*N*_*Ag*_) at H1–H3, mean N in the tuber biomass (*N*_*Tuber*_) at H2 and at H3; and the mean plant N content during the entire growth period (*N'*) in the potato lines grown in the field (2015) and greenhouse experiment (2014).

	**Field**		**Greenhouse**			
	**Dinamo**	**T-2012**	**Kuras**	**T-8003**	**Dinamo**	**T-2012**
N in initial seed (*N_*S*_*)	0.10 ^A^^*^ (0.010)^**^	0.05 ^B^ (0.002)	0.07 (0.001)	0.07 (0.001)	0.07 (0.002)	0.06 (0.001)
*Total plant N at H1*	1.65 (0.25)	1.04 (0.18)	0.50 ^a^ (0.07)	0.04 ^ab^ (0.09)	0.32 ^ab^ (0.03)	0.20 ^b^ (0.04)
*Total plant N at H2*	3.97 (0.64)	2.53 (0.37)	1.66 (0.08)	1.62 (0.05)	1.77 (0.08)	1.78 (0.07)
*Total plant N at H3*	3.56 (0.25)	4.33 (0.49)	4.37 ^b^ (0.34)	5.03 ^ab^ (0.35)	5.12 ^ab^ (0.58)	6.51 ^a^ (0.73)
*N_*Ag*_* at H1	1.65 (0.25)	1.05 (0.18)	0.49 ^a^ (0.06)	0.43 ^ab^ (0.09)	0.31 ^ab^ (0.03)	0.20 ^b^ (0.04)
*N_*Ag*_* at H2	1.63 (0.31)	1.21 (0.20)	1.37 ^b^ (0.07)	1.47 ^ab^ (0.05)	1.59 ^a^ (0.09)	1.66 ^a^ (0.07)
*N_*Ag*_* at H3	0.45 ^B^ (0.07)	0.50 ^A^ (0.07)	1.50 ^c^ (0.05)	1.70 ^bc^ (0.15)	1.73 ^b^ (0.06)	2.22 ^a^ (0.16)
*N_*Tuber*_* at H2	2.33 ^A^ (0.33)	1.32 ^B^ (0.25)	0.28 ^a^ (0.02)	0.14 ^b^ (0.02)	0.17 ^b^ (0.02)	0.12 ^b^ (0.01)
*N_*Tuber*_* at H3	3.10 (0.21)	3.83 (0.44)	2.87 (0.31)	3.33 (0.29)	3.38 (0.59)	4.28 (0.65)
Mean plant N (*N'*)	3.34 (0.32)	2.80 (0.31)	1.54 (0.03)	1.40 (0.08)	1.46 (0.05)	1.52 (0.05)

*The unit for all the values is g plant^−1^*.

* *Results of Tukey HSD test at α = 0.05*.

** *Standard error (n = 5 in greenhouse; n = 10 in field)*.

*Different letters indicate statistically significant differences between groups*.

**Table 4:** Means of dry and fresh above-ground (*Ag*) and tuber biomass (g plant^−1^) at different developmental stages, and tuber amylose content (g plant^−1^) in “Dinamo” and “T-2012” and TAG content (nmol plant^−1^) in “Kuras” and “T-8003,” grown in the field (2015) and greenhouse experiment (2014).

	**Field**		**Greenhouse**			
	**Dinamo**	**T-2012**	**Kuras**	**T-8003**	**Dinamo**	**T-2012**
Dry *Ag* biomass at tuber initiation	28.23 ^A^^*^ (3.31)^**^	18.91 ^B^ (2.80)	7.12 ^a^^*^ (1.00)	6.22 ^ab^ (1.22)	4.60 ^ab^ (0.34)	3.22 ^b^ (0.50)
Dry *Ag* biomass at flowering	65.15 (12.43)	50.15 (7.79)	51.28 (1.38)	48.92 (0.95)	48.20 (1.80)	50.82 (1.56)
Dry *Ag* biomass at final harvest	33.03 (3.12)	35.07 (7.10)	62.51 (1.87)	66.32 (2.79)	66.23 (2.80)	77.09 (5.43)
Fresh *Ag* biomass at final harvest	55.95 ^B^ (10.19)	111.74 ^A^ (14.51)	386.9 ^c^ (18.06)	425.3 ^b^ (6.73)	446.2 ^ab^ (12.54)	460.6 ^a^ (3.92)
Dry tuber biomass at flowering	255.29 ^A^ (35.57)	156.19 ^B^ (35.57)	36.86 ^a^ (3.61)	12.52 ^c^ (1.82)	20.02 ^b^ (2.02)	10.57 ^c^ (1.47)
Fresh tuber biomass at flowering	462.08 (71.54)	293.72 (40.90)	149.74 ^a^ (17.27)	89.83 ^b^ (13.38)	98.13 ^b^ (7.96)	80.66 ^b^ (11.59)
Tuber amylose or TAG^†^ content (Menzel et al., 2015; Hofvander et al., 2016)	60.34 ^B^ (4.90)	117.88 ^A^ (14.22)	61.53^†^^b^ (35.26)	500.4^†^^a^ (50.67)	14.31 ^a^ (2.29)	11.54 ^a^ (3.07)

* *Results of Tukey HSD test at α = 0.05*.

** *Standard error (n = 5 in greenhouse; n = 10 in field)*.

† *Triacylglycerol*.

*Different letters indicate statistically significant differences between groups*.

**Figure 2:**
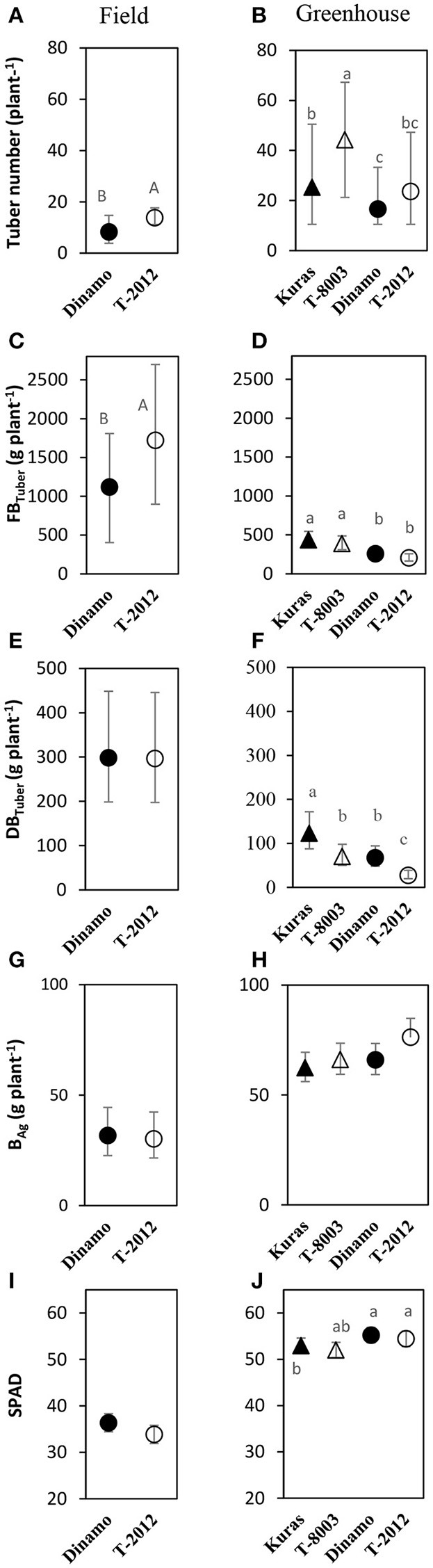
Means of tuber number, tuber yield and biomass in the field experiment (**A,C,E,G,I**; 2015) and greenhouse (**B,D,F,H,J**; 2014). Tuber number, *FB*_*Tuber*_ = fresh tuber biomass, *DB*_*Tuber*_ = dry tuber biomass and *B*_*Ag*_= dry above-ground weight are measured at H3; SPAD= leaf chlorophyll content at H2. Two GM lines (“T-8003” and “T-2012”) and their parents (“Kuras” and “Dinamo,” respectively) were grown in the greenhouse, and “T-2012” and “Dinamo” were grown in the field experiment. Related potato lines are shown with a similar marker. Parental varieties are shown by filled markers and GM lines are shown by open markers. Error bars indicate 95% confidence intervals, letters show results of Tukey HSD test at α = 0.05.

At final harvest, “T-2012” had nearly twice as many tubers and 40% higher fresh tuber biomass per plant (*FB*_*Tuber*_) than its parent “Dinamo,” but their *DB*_*Tuber*_ was similar (Table 5; Figure 2). Thus, both potato lines allocated the same amount of carbon to the total tuber harvest, but “T-2012” allocated more water to the tubers.

**Table 5:** ANOVA for number of tubers and above-ground and tuber biomass in the greenhouse (2014) and field experiment (2015).

**Source of variation**		**Tuber number**		***FB_Tuber_***		***DB_*Tuber*_***		***B_*Ag*_***		**SPAD**	
	**df**	***F***	***P***	***F***	***P***	***F***	***P***	***F***	***P***	***F***	***P***
**FIELD EXPERIMENT**											
Line^*^	1	5.44	**0.035**	4.55	**0.049**	0.01	0.968	0.07	0.797	3.41	0.072
**GREENHOUSE EXPERIMENT**											
Line^**^	1	5.44	**0.035**	4.55	**0.049**	0.01	0.968	0.07	0.797	3.41	0.072
Within line types^*^	2	12.35	**<0.001**	1.45	0.260	10.84	**0.001**	2.38	0.120	0.42	0.660
Between line types^***^	1	31.77	**<0.001**	29.33	**<0.001**	21.84	**<0.001**	3.90	0.065	7.06	**0.017**

*The FB_Tuber_, fresh tuber biomass; DB_Tuber_, dry tuber biomass; B_Ag_, dry above-ground biomass, are measured at final harvest (H3); and SPAD, leaf chlorophyll content at the flowering stage (H2)*.

* *Comparison between “Dinamo” and “T-2012” lines*.

** *Comparisons between each of the GM lines and their parental relatives (i.e., “Kuras” vs. “T-8003,” “Dinamo” vs. “T-2012”)*.

*** *Comparison between the oil and amylose potato lines*.

In terms of *NUE* and its components, the N uptake or recovery efficiency from soil *(NUpE)* was 20% higher in “T-2012” compared to its parental “Dinamo,” whilst the biomass-based N utilization efficiency *(NUtE)* was 15% lower, and the overall *NUE* was similar between the two lines (Table 6). When the calculations of N utilization efficiency and *NUE* were based on the amylose yield, the GM-line had 60% higher utilization efficiency (*NUtE*_*amylose*_) and twice as high N use efficiency (*NUE*_*amylose*_) compared to the parental line.

**Table 6:** The N use efficiency and its components calculated according to Moll et al. (1982) using the data from the potato GM-line “T-2012” and its parent (“Dinamo”) grown in the field experiment.

**Potato line**	***NUpE***	***NUtE***	***NUE***	***NUtE_*amylose*_***	***NUE_*amylose*_***
“Dinamo”	0.73	86.4	62.8	17.0	12.3
“T-2012”	0.88	73.6	65.0	27.2	24.1

The N uptake efficiency (NUpE) is calculated as the crop N content at maturity divided by the soil available N supply including fertilizer; the N utilization efficiency (NUtE and NUtE_amylose_) is calculated as the tuber biomass respective amylose yield divided by the crop N content (incl. tuber N content) at maturity; and NUE = NUpE × NUtE or NUE_amylose_ = NUpE × NUtE_amylose_. The soil N supply is here calculated as the sum of assumed soil N availability before fertilization (20% of 95 kg N ha^−1^ was here assumed plant available, i.e., 19 kg N ha^−1^) and the applied fertilizer (177 kg N ha^−1^). All units are g g^−1^

**Figure 3:**
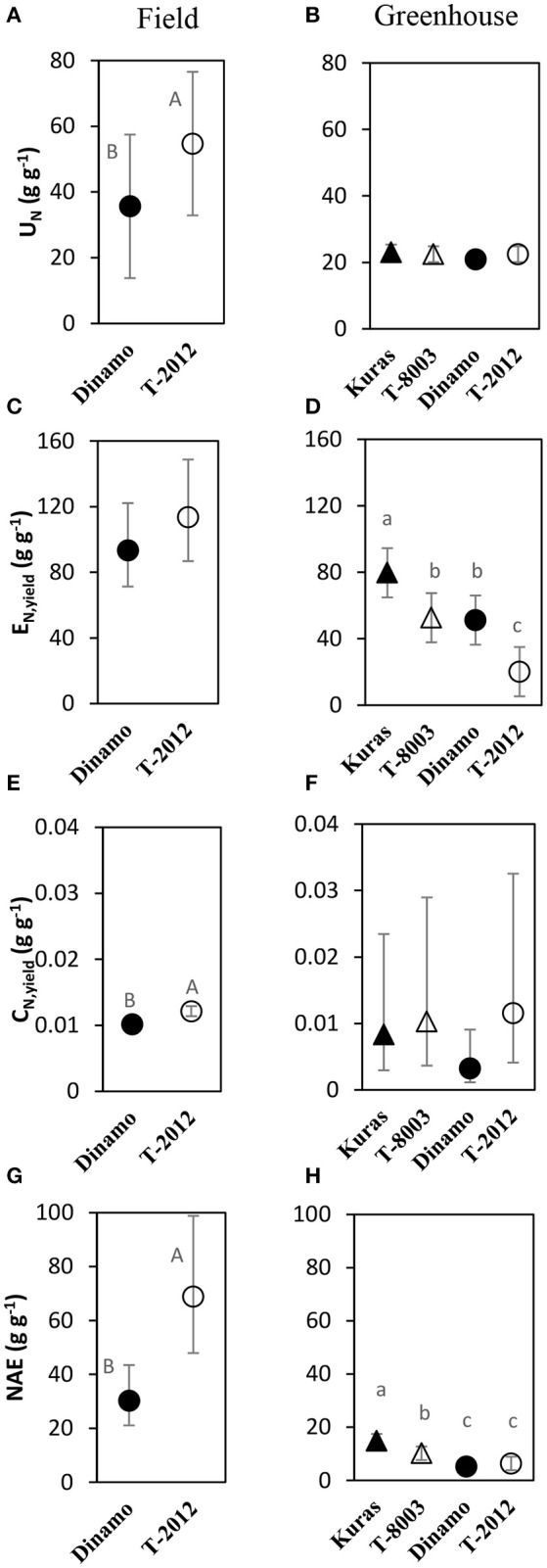
Means of N accumulation efficiency and its components in the field experiment (**A,C,E,G**; 2015) and greenhouse (**B,D,F,H**; 2014). *U*_*N*_ = N uptake efficiency, *E*_*N,yield*_= yield specific N efficiency, *C*_*N,yield*_ = yield N concentration, *NAE* = N accumulation efficiency. Two GM lines (“T-8003” and “T-2012”) and their parents (“Kuras” and “Dinamo,” respectively) were grown in the greenhouse, and “T-2012” and “Dinamo” were grown in the field experiment. The calculations are based on total tuber dry weight per plant and according to Table 2. Related potato lines are shown with a similar marker. Parental varieties are shown by filled markers and GM lines are shown by open markers. Error bars indicate 95% confidence intervals, letters show results of Tukey HSD test at α = 0.05.

By means of *NAE* and its components, we found clear effects of the genetic modification (Table 7). Thus, despite of similar *DB*_*Tuber*_, “T-2012” had 53% higher *U*_*N*_ and 20% higher *C*_*N,yield*_ than “Dinamo,” which resulted in a strong increase (+120%) of the overall *NAE* in the GM line “T-2012” compared with its parent “Dinamo” (Figure 3). The GM potato line started out with a lower initial N content in the seed tubers compared to the parental variety, but compensated for the initially lower N content by continued N uptake especially between flowering and maturity, a period in which N accumulation in “Dinamo” had stagnated (Figure 1; Table 3). The SPAD values measured at flowering, *B*_*Ag*_ at final harvest and *E*_*N,yield*_ were similar between “T-2012” and “Dinamo” (Figures 2, 3; Tables 4, 6). In general, the desired end product of the field-grown amylose GM line was produced in much smaller amounts by the parental non-GM variety (Table 4), and the N accumulated by the end of the growing season was similar in the GM line and its non-GM parental variety (e.g., Figure 1A). Technically, the calculation of *E*_*N,yield*_ in terms of end product, e.g., amylose accumulation per unit of mean plant N in the field experiment, would therefore result in much higher values for the GM line compared to the parental line.

**Table 7:** ANOVA for N accumulation efficiency and its components in the field (2015) and greenhouse (2014).

**Source of variation**		***U_*N*_***		***E_*N,yield*_***		***C_N,yield_***		***NAE***		***N'***	
	**df**	***F***	***P***	***F***	***P***	***F***	***P***	***F***	***P***	***F***	***P***
**FIELD EXPERIMENT**											
Line^*^	1	4.81	**0.040**	1.38	0.259	17.40	**<0.001**	15.44	**<0.001**	1.66	0.210
**GREENHOUSE EXPERIMENT**											
Within line type^**^	2	0.42	0.666	8.67	**0.003**	1.76	0.020	4.03	**0.038**	2.00	0.168
Between line types^***^	1	7.06	**0.017**	19.01	**<0.001**	0.73	0.407	31.22	**<0.001**	0.20	0.660

* *Comparison between “Dinamo” and “T-2012” lines*.

** *Comparisons between each of the GM lines and their parental relatives (i.e., “Kuras” vs. “T-8003,” “Dinamo” vs. “T-2012”)*.

*** *Comparison between the oil and amylose potato lines*.

*U_N_, N uptake efficiency; E_N,yield_, yield specific N efficiency; C_N,yield_, yield N concentration; NAE, N accumulation efficiency; N', plant mean N content during the entire growth period. Dry tuber biomass (DB_Tuber_) at final harvest (H3) was used for the calculations*.

### Greenhouse experiment

The N amount in initial biomass (*N*_*S*_), i.e., plant cuttings, was similar in all potato lines (average value of 0.07 g g^−1^, Table 3). At flowering stage (H2), the dry tuber biomass per plant was two to three times higher in the parental varieties “Kuras” and “Dinamo” than the GM potato lines “T-8003” (oil potato) and “T-2012” (amylose potato), whereas their above-ground dry weight was similar (Table 4).

At final harvest (H3), the average number of tubers per plant was 1.5 to two times higher in the GM lines than their corresponding parents, while the tuber dry biomass (*DB*_*Tuber*_) and the yield specific N efficiency (*E*_*N,yield*_) were 1.5–2.5 times higher in the parental varieties than in the GM lines (Figures 2, 3; Tables 4, 6, within line type effect). Tuber fresh biomass (*FB*_*Tuber*_) and tuber N concentration (*C*_*N,yield*_) at maturity, total plant above-ground biomass (*B*_*Ag*_), SPAD at anthesis and N uptake efficiency (*U*_*N*_) were all similar between the GM lines and their respective parents (Figures 2, 3; Tables 4, 6, within line type effect). Thus, the overall pattern was a similar *U*_*N*_ and *C*_*N,yield*_, but a 50% lower *E*_*N,yield*_ in the GM lines.

### Comparison between field and greenhouse experiment

Seed tubers were planted in the field experiment whilst in the greenhouse, the GM lines and parental varieties were planted as cuttings; this difference influenced the results especially regarding the comparison between the two experimental set-ups. In terms of *NAE* and its components, the differences in the initial plant material between the greenhouse (cuttings) and field (seed potato) are fully accommodated. At tuber initiation, the average above-ground biomass per plant was higher in plants grown in field than in the greenhouse (Table 4). At final harvest stage, the mean number of tubers and the above-ground biomass per plant (*B*_*Ag*_) were greater in the greenhouse experiment than in the field, while the tuber numbers as well as biomass (fresh and dry) was greater in the field (Figure 2). On average, the potato plants grown in the field had higher N uptake efficiency (*U*_*N*_), yield specific N efficiency (*E*_*N,yield*_), plant mean N content (*N'*) and *NAE* than the lines grown in the greenhouse (Figure 3, Table 3). In the greenhouse conditions, the N accumulation followed an exponential pattern throughout the entire experimental period (Figure 1B), while the biomass increased at diminishing returns during the study period (Figure 1D). In the field, the N and biomass accumulation showed a similar and clear seasonal pattern, with strong and exponential accumulation early in the growing season and much slower (GM line) or stagnating (non-GM line) accumulation late in the season (Figures 1A,C).

## Discussion

### Growth and yield

Potato tubers almost exclusively accumulate starch as energy storage product; a carbohydrate composed of a mixture of amylose and amylopectin to a ratio of ~1:4. Increased amylose to starch ratio in potato has been achieved by reducing amylopectin synthesis, e.g., branching of the amylopectin, through the inhibition of two starch branching enzyme genes *(SBE)* (Andersson et al., 2006). In a study by Hofvander et al. (2016), a GM potato line was developed by expressing a transcription factor (*WRINKLED1*), resulting in tubers which accumulate starch as well as oil (TAG). Previous field and greenhouse studies of lines with these traits have indicated increased tuber yield as compared with corresponding parental varieties (Menzel et al., 2015; Hofvander et al., 2016). Based on those studies, we hypothesized that the high-amylose and oil GM potato lines would produce more tuber yield than their parental non-GM relatives by the end of their growth period. This hypothesis was partially supported in the field experiment where the fresh tuber yield at final harvest was greater in a high-amylose GM line than its parent “Dinamo.” According to these previous studies (Menzel et al., 2015; Hofvander et al., 2016), the modifications for initiated oil synthesis and increased amylose accumulation in potato tubers are both associated with a lower starch content in the tubers, which is the main component of dry matter (Pinhero et al., 2016). A decreased carbon allocation to storage in the high-amylose and oil potato lines can result in increased levels of soluble sugars in the tubers, i.e., fructose, glucose and sucrose in the oil potato tubers and fructose and glucose in the high-amylose tubers. Accordingly, it can be speculated that the altered sugar levels in GM potato lines might stimulate an osmosis mechanism allocating more water to the tubers. In contrast to the fresh tuber biomass, the dry tuber biomass did not differ between the lines in the field, and was lower in the GM lines than their parental varieties under greenhouse conditions.

Despite having significantly different fresh tuber yields, the above-ground biomass at the final harvest was similar in the GM lines and their parental varieties when grown both in the field and in the greenhouse. In fact, the parental variety “Dinamo” produced more above-ground biomass at the establishment stage (H1) than the GM line “T-2012,” which along with an altered starch accumulation in “T-2012” tubers (in agreement with Menzel et al., 2015), may explain why “Dinamo” had a higher tuber dry biomass at the flowering stage (H2). Thus, it appears that in the field, the GM line “T-2012” produced a higher fresh tuber yield by the end of the growth period with a lower above-ground biomass throughout the whole growth period. It is possible that the GM line “T-2012” allocated more resources to below-ground biomass, which could have resulted in a below-ground competitive advantage at the early growth stage and therefore the production of equal dry tuber biomass along with increased fresh tuber yield compared to its parent. This idea is supported by the results from the greenhouse experiment, which showed that at the tuber initiation stage (H1), the GM lines had a higher below-ground biomass (root and stolon) than their parents (mean values of 2.9, 4.2, 2.4, and 4.0 g plant^−1^ for “Kuras,” “T-8003,” “Dinamo,” and “T-2012,” respectively; data not shown). In agreement to our results, Iwama (2008) concluded that an early below-ground establishment is of vital importance for final tuber production in potato varieties. Thus, the higher number of tubers and the higher fresh tuber yield in the GM potato line compared to its parent in our field study were likely the results of greater root and stolon growth early in the growing season, and an altered carbon allocation leading to an increased water ratio after the flowering stage.

Several leaf functional traits, especially when assessed at the flowering stage, are frequently correlated to yield in various crops including wheat (Boissard et al., 1993), barley (Spaner et al., 2005), grass species (Gaborcik, 2003) and potato (Zheng et al., 2015); and can therefore be used for, e.g., tuber yield prediction in potato crops. Thus, easy to assess functional traits such as leaf chlorophyll content via SPAD measures, could facilitate the breeding in order to identify targeted crop traits associated with the yield production (Poorter and Garnier, 1999). In our field and greenhouse studies, the SPAD values measured at the flowering stage did not differ between the GM lines and their parental relatives, showing that SPAD values assessed at flowering were not indicative of fresh tuber yield in the potato lines assessed here.

### Nitrogen economy

In general, our values of *NUE* and its components were similar to those reported in the literature for potato grown under similar conditions than ours, e.g., Zebarth et al. (2004). We hypothesized that an increased yield in GM potato lines is associated with an increase in N uptake efficiency (*NUpE, U*_*N*_), N utilization efficiency (*NUtE*) and/or yield specific N efficiency (*E*_*N,yield*_). In the field study, the total plant N content increased more in the GM line “T-2012” than its parent “Dinamo,” especially during the period between flowering and maturity. A higher N uptake efficiency in “T-2012” compared to “Dinamo” is particularly apparent when considering the lower N content in its initial biomass (by factor 0.5) in the calculation of *U*_*N*_, but also in the calculation of N uptake efficiency in terms of the amount of N recovered from soil *(NUpE)*. The calculations according to Weih et al. (2011) integrated the N pool of the entire growing season and revealed clearly higher N uptake efficiency and overall *NAE* in the GM-line compared to its parent (Figure 3). In addition, the large difference in seed potato size (and thereby the size of the initial plant N pool) between the GM-line and its parent is not considered in the calculation of *NUpE*, but accommodated in the calculation of *U*_*N*_. The higher *U*_*N*_ in the “T-2012” compared to its parent was probably facilitated by its greater root development early in the growing season, as discussed above.

In the field experiment, the biomass-based N utilization efficiency (*NUtE*) was lower in the GM-line compared with its parent, while the yield-specific N efficiency (*E*_*N,yield*_) tended to be higher in the GM-line. Both *NUtE* and *E*_*N,yield*_ quantify the tuber biomass productivity per plant N. However, the calculation of *E*_*N,yield*_ is based on the mean plant N pool during the entire period of tuber production, while *NUtE* considers only the final tuber N pool at maturity (assuming that final N pool would be available during the entire period of tuber production). Therefore, the *E*_*N,yield*_ here probably better reflects the tuber productivity per plant N compared to *NUtE*. The higher amylose-based N utilization efficiency (*NUtE*_*amylose*_) in the GM-line compared to its parent (Table 6) clearly shows the potential of this line to generate higher amounts of the targeted end product per plant N also in terms of *NUE* and its components. In contrast to the field, the *E*_*N,yield*_ was lower in the GM lines than their parents in the greenhouse experiment. The partly differential *E*_*N,yield*_ pattern in the greenhouse compared to the field condition could be related to the generally lower *U*_*N*_ along with higher SPAD values observed in the greenhouse, which is expected to affect N economy (Asplund et al., 2014; Zheng et al., 2016). The different genotype responses in *U*_*N*_ and *E*_*N,yield*_ between the growing conditions suggests that genotype by environment interaction was important here. Thus, in line with our hypothesis, a high fresh tuber production in the high-amylose GM line grown in the field study was mainly the result of an increased *NUpE* or *U*_*N*_. According to the field study, an altered carbon allocation in the GM potato line “T-2012” with increased amylose content in tubers, may have resulted in an improved N economy in terms of the N accumulation in the critical developmental stages for yield production, e.g., post-flowering, as well as a slight (but not statistically significant) increase in *E*_*N,yield*_.

In the field study, “T-2012” had a higher N re-translocation to the harvested tuber (tuber N content, *C*_*N,yield*_), which along with a higher *U*_*N*_ and *E*_*N,yield*_, resulted in a greater overall *NAE* in “T-2012” than its parent “Dinamo.” This means that both the growing-season N uptake and the N accumulation in the harvested tuber per unit of N in the initial seed tuber were higher in the GM potato line “T-2012.” Overall, the changed quality in the GM potato line was associated with higher yield, but also higher N accumulation in the crop and the tubers; and thereby greater potential N removal from the production system when the tubers are harvested. In fact, despite only half of the N amount was added to the agro-ecosystem by the seed tubers of the GM line compared to its parent, 18% more N was removed from the system by the harvested GM tubers (differences in total plant N content at final harvest; Table 3), suggesting a c. 25% greater net N export from an agro-ecosystem with the GM-line compared with its parent. This indicates that the cultivation of the GM line in the long term could require higher N fertilization rates as compared to its parent in order to replace the additional N removed from the production system by the GM potato. Given that these potato lines are cultivated for their desired traits, i.e., the induced oil accumulation or enhanced amylose content, the evaluation of ratios between the amounts of produced amylose or oil and the N amount taken up from the soil (e.g., *NUtE*_*amylose*_, Table 6) and potentially removed from the production system by the crop harvest appears to be a useful tool to estimate the potential impacts of growing these GM potatoes on the N cycle. For example in our field study, the estimated amount of amylose produced per unit of plant-N that would be removed by the plant harvest, was higher in “T-2012” than in “Dinamo” (mean values of 27.2 and 16.9 g g^−1^ for “T-2012” and “Dinamo,” respectively). This means that, despite potentially removing more plant-N from the production system, the GM line “T-2012” produced more amylose per unit of plant-N taken up and potentially removed from the production system; and therefore can be regarded as more N efficient for amylose production. In addition, crops with improved N uptake efficiency, such as the high-amylose potato line in our study, can in some cases be important complementary measures for reducing N losses to water, provided that the N application rate does not exceed the enhanced N uptake potential of the N-efficiency-improved crops (Tidaker et al., 2016). Nevertheless, any ecosystem scale effects of growing these GM potatoes on the N removal and cycling need to be investigated by assessing the N flows at ecosystem scale and in the long term (e.g., several years).

Apart from gaining insights into the effects of genetic modification on non-target physiological traits and processes (here N economy), the differences in the N economy of the GM amylose potato (compared to its parent) found here have implications for commercial starch (or oil) potato production, because they affect the N fertilization requirements of the crop and the tuber (or starch/oil) productivity per plant N. In a commercial perspective, both the fertilization requirement and the starch (or oil) productivity per unit of N fertilizer required are important factors for the evaluation of the economic and environmental performance of the crop, especially when different crops are compared. In a bio-economy perspective, starch and oil crops are key sources for the sustainable production of biomass (Zeeman et al., 2010; Yadava et al., 2012), and the N fertilization requirements together with starch productivity per N required need to be compared across the various crops that potentially could be used for producing the same type of biomass. Assessments of N economy with the methods used here (Moll et al., 1982; Weih et al., 2011) could provide important information for the comparison across crops when combined, as these methods are flexible, applicable to various crops and show different aspects of their N economy.

### Effect of growth condition on the variation of yield and nitrogen economy

We hypothesized that the effect of modified traits for an increased amylose and oil tuber content on the non-targeted traits would be similar in different environmental conditions. In contrast to our expectation, the observed patterns in the non-targeted traits assessed here (tuber yield and N economy traits) and between the GM-lines and their parental varieties, differed when plants were grown in two different conditions, i.e., field and greenhouse. Environmental influences during growth have been shown previously to strongly affect plant metabolism in GM plants (e.g., Baker et al., 2006), and these environmental influences apparently also affected the growth responses in our comparison between potato GM-lines and their parental lines. The significant differences between the GM lines and their parents in their fresh tuber biomass and N uptake efficiency observed in our field study were not apparent in the greenhouse experiment. Moreover, the mean plant N during the entire growth period (*N'*) and the mean N uptake efficiency across potato lines were greater in the field than in the greenhouse (both by factor 2.1); which is similar to the observations reported for wheat (Asplund et al., 2014). A low amount of substrate per unit of plant biomass in the pot as compared to the field condition may have limited the root development and consequently depressed the overall N uptake during the entire growth period of the plants grown in the greenhouse (Timlin et al., 2006; Poorter et al., 2012). In contrast to the greenhouse, the GM line “T-2012” in the field took advantage of its greater N accumulation capacity especially late in the growing season, and produced significantly higher fresh tuber yield than its parent. As a possible consequence of the most likely limiting effect of the growing containers on N uptake and tuber development, the mean tuber yields in the greenhouse were lower than in the field; an observation similar to Bones et al. (1997).

In contrast to the tuber yield, the mean above-ground biomass was greater in the greenhouse than in the field, which can be ascribed to the low light irradiance coupled with a higher temperature in the greenhouse than in the field (Bones et al., 1997). In concordance with our results, Timlin et al. (2006) showed that higher temperatures negatively affected potato tuber development, while at the same time stimulated above-ground biomass production. Similarly, and in agreement with the other studies on various plants (Wang et al., 2007; Ibrahim and Jaafar, 2011; Asplund et al., 2014), the SPAD values were greater in greenhouse than in the field. It appears that the greenhouse conditions, with a low-light/high-temperature climate, a limiting effect of the pot on the below-ground growth of the plants, and the much less pronounced seasonality of growth conditions (cf. Figure 1 for the temporary pattern of N accumulation), resulted in altered carbon and N allocation pattern compared to those in the field. Our results from the comparison between greenhouse and field conditions suggest that the growth and N economy results from greenhouse conditions are not necessarily representative for potato growth and N economy under field conditions.

In conclusion, the effect of cultivation conditions clearly influenced the growth and whole-plant N economy traits assessed here. For example, the final fresh above-ground plant biomass and N pool were considerably higher in the greenhouse conditions, whilst the tuber yield and growing-season mean plant N pool were higher in the field conditions. The strong environmental effects on the expression of non-target traits needs to be considered in the comparison of GM and non-GM plants, and any results from greenhouse experiments should be verified in field settings. The higher tuber yield in the field-grown high-amylose potato line was associated with changed N economy as reflected by a higher N uptake efficiency (*sensu NUpE* and *U*_*N*_), tuber N content and N accumulation efficiency (*NAE*). Due to a more than 50% increase in N uptake efficiency (*sensu U*_*N*_) compared to the parental variety, the cultivation of the high-amylose line is expected to require higher N fertilization rates, but also to allow a more N-efficient production of amylose not only per land area but also per unit N in the plant and soil (i.e., higher *NUtE*_*amylose*_ and *NUE*_*amylose*_).

## Datasets are available on request

The raw data supporting the conclusions of this manuscript will be made available by the authors, without undue reservation, to any qualified researcher.

## Author contributions

MW and MA planned the research and, together with FP, designed the experiments. FP conducted the sampling from the greenhouse and field trials, and did most of the data analysis. FP wrote major parts of manuscript, with substantial inputs from MW and MA during the writing.

### Conflict of interest statement

The authors declare that the research was conducted in the absence of any commercial or financial relationships that could be construed as a potential conflict of interest.
